# Offspring Long-Term Respiratory Morbidity Following Cesarean Delivery at Different Stages of Labor

**DOI:** 10.3390/jcm15051827

**Published:** 2026-02-27

**Authors:** Gil Gutvirtz, Hagar Brami, Tamar Wainstock, Eyal Sheiner

**Affiliations:** 1 Department of Obstetrics and Gynecology, Soroka University Medical Center, Beer-Sheva 84101, Israel; gilgut@bgu.ac.il (G.G.); hagar.mashal@gmail.com (H.B.); 2 Department of Public Health, Faculty of Health Sciences, Ben-Gurion University of the Negev, Beer-Sheva 84105, Israel; wainstoc@bgu.ac.il

**Keywords:** pregnancy, cesarean delivery, non-progressive labor, dystocia, vaginal flora, offspring, respiratory morbidity

## Abstract

**Background/Objectives**: Cesarean deliveries (CD) have been associated with an increased risk for offspring long-term respiratory morbidity. We sought to evaluate whether children born via CD in different stages of labor, and partially exposed to vaginal flora, would differ in their long-term respiratory morbidity. **Methods**: A population-based study comparing long-term respiratory morbidity of children according to their mode of delivery and CD indication was conducted. Children born via CD for first stage non-progressive labor (CD-NPL1) and children born via CD for non-progressive labor in the second stage (CD-NPL2) were compared with children born vaginally (VD) as a reference group. Offspring hospitalizations up to 18 years involving respiratory morbidities were evaluated. **Results**: 313,782 deliveries were included; 97.7% were VD, 1.6% were CD for NPL1 and 0.7% were CD for NPL2. The overall respiratory-related hospitalization rates as well as the cumulative incidence of respiratory hospitalizations were significantly higher in children born via CD, with a graded association, related to fetal exposure to vaginal flora, noted between VD, CD-NPL2 and CD-NPL1 with the highest incidence. In a Cox model, controlling for multiple confounding variables, NPL1 (vs. VD) was associated with an increased risk for offspring long-term respiratory morbidity (aHR 1.15), while NPL2 did not differ in risk. **Conclusions**: The risk for respiratory morbidity is increased for NPL1 offspring (with lower exposure to vaginal flora) as compared with NPL2 and VD offspring, with a graded association noted between exposure to vaginal flora during labor and the risk for offspring long-term respiratory morbidity.

## 1. Introduction

For many years, the human fetus was thought to develop within a bacteria-free environment. However, several recent studies have reported the presence of microbial DNA in the placenta, amniotic fluid and meconium, suggesting that the fetus may be exposed to microorganisms before delivery [1,2]. Nevertheless, it is evident that upon delivery, the maximal fetal exposure to the maternal microbiome occurs during the passage through the birth canal, making the mode of delivery the most important factor to set the pattern of fetal airway and GI tract colonization [3,4,5]. Early life gut microbiota, in turn, has been increasingly recognized as major contributors to short and long-term human health and diseases [6]. Consequently, it has been hypothesized that neonatal exposure to maternal vaginal flora may reduce the risk of the offspring to develop infectious and allergic disorders. For example, numerous studies have found that children born via elective cesarean delivery (CD), unexposed to maternal vaginal flora, were at higher risk for infectious [7,8] and respiratory [9] morbidity, including asthma [10], bronchiolitis [11] and allergic rhinitis [12]. Other studies on children born after elective CD also elucidated their heightened risk for other various long-term health complications including cardiovascular [13], neurological [14] and gastrointestinal morbidity [15]. While most of these studies focused on children born following elective CD, thus investigating children unexposed to maternal vaginal flora, those who intended to explore unscheduled CD were unable to account for the CD indication and stage of labor (whether first or second) when the CD was performed. In 2006, Gerten et al. [16] conducted a population-based case-control study and found that CD was an independent risk factor for neonatal respiratory distress syndrome (RDS), particularly if the cesarean was performed in a nonlaboring patient. However, their definition of laboring women was based on variables that positively indicated that labor had occurred, without the possibility to discern the different stages of labor the women were finally delivered by cesarean. Interestingly, Azad et al. [17] found the lowest microbial richness and diversity among elective CD infants as compared with those delivered vaginally or by CD after the onset of labor. We consider it plausible that fetal exposure to maternal vaginal flora varies across different stages of labor, potentially influencing airway and gastrointestinal tract colonization and affecting susceptibility to respiratory disease later in life. Therefore, we conducted this study to examine the long-term respiratory morbidity of offspring born via vaginal delivery, representing maximal exposure to maternal vaginal flora, compared to those delivered by cesarean section at different stages of labor, with presumed partial exposure.

## 2. Materials and Methods

This population-based cohort analysis included all singleton deliveries that took place during an almost 30-year-long (between January 1991 and December 2019) study period in a single medical center (Soroka University Medical Center (SUMC)), which is the only tertiary hospital located in the southern district of Israel, with more than 17,000 births annually. The study is derived from a non-selective population, as medical care in Israel is provided under the National Health Insurance Law and is universally accessible to all citizens, including obstetric care and hospitalizations. Importantly, the southern region of Israel is characterized by positive migration rate which assumes most children born in our institute will also be hospitalized in this institution, if needed, as no other hospitalization facilities are available in the region. This allows for a long-term follow-up on offspring born to mothers that delivered in SUMC.

Multiple gestations, fetuses with congenital malformations or chromosomal abnormalities, and cases of perinatal mortality were excluded from the study. Cesarean deliveries due to intrapartum complications including non-reassuring fetal heart rate (NRFHR) tracings, placental abruption, cord prolapse or failed instrumental delivery leading to an emergency CD were also excluded from the study, which enabled us to focus only on CDs performed for labor dystocia in the first or second stage of labor.

The primary exposure was mode of delivery (VD vs. CD) and the specific indication for CD. We compared children delivered by CD for non-progressive labor in the first (NPL1) and second stage (NPL2) with those born vaginally (VD) who served as the reference group. Both NPL1 and NPL2 diagnoses were obtained from the obstetrical documentation, based on the diagnosis given during labor by the attending obstetrician. The primary outcome included any respiratory-related hospitalization of the offspring up to the age of 18 years, collected using a pre-defined list of ICD-9 codes detailed in the Supplementary Table (Table S1), used in SUMC pediatric wards. Follow-up of the offspring was terminated upon the first hospitalization at SUMC involving a respiratory illness (i.e., an event) or until censored. Censoring occurred when the subject reached the age of 18 years (by calculation from date of birth), end of the study period (December 2019) or upon death during hospitalization for non-respiratory morbidity.

The data for this study was retrieved from two independently maintained electronic datasets that were subsequently linked based on maternal and offspring ID numbers: (1) the perinatal registry of the Department of Obstetrics and Gynecology; and (2) the pediatric hospitalization registry; both available through SUMC archives. The perinatal database contains detailed maternal demographic and obstetric data recorded at the time of admission for labor and immediately after delivery by the attending obstetrician. The pediatric database documents demographic characteristics and ICD-9 diagnostic codes for all admissions to the pediatric wards. Prior to archive, trained medical secretaries systematically verify the information to enhance data integrity and completeness. Diagnostic coding is assigned after comprehensive review of prenatal records and standard hospital documentation.

Statistical analyses were conducted using SPSS software Version 29 (IBM/SPSS, Armonk, NY, USA). Categorical variables were compared using the chi-square test for general association, and continuous variables with normal distribution were analyzed using the independent samples *t*-test. Kaplan–Meier survival curves were generated to evaluate cumulative respiratory-related morbidity over time among exposed groups (CD) and the unexposed group (VD). Differences between groups were assessed using the log-rank test. A multivariable Cox regression model was applied to evaluate whether mode of delivery and the specific indication for cesarean delivery were independently associated with the risk of respiratory-related hospitalizations in offspring up to 18 years of age, after adjustment for potential confounding variables including maternal age, nulliparity, maternal obesity, smoking, use of fertility treatments, diabetes mellitus, hypertensive disorders, gender, ethnicity, birthweight and child’s year of birth. Missingness across the variables was minimal (ranging from 0 to 865), and therefore did not prohibit their inclusion in the model. A *p* value of <0.05 was considered statistically significant.

During the preparation of this work, the authors utilized ChatGPT based on GPT-4.1 for spelling and rephrasing. After employing this tool/service, the authors thoroughly reviewed and edited the content as needed, and take full responsibility for the final publication.

## 3. Results

A total of 313,782 deliveries met the inclusion criteria, of which 97.7% were VD, 1.6% were CD for NPL1, and 0.7% were CD for NPL2. Table 1 presents selected maternal demographic characteristics across the three groups. Compared to those who had VD, individuals who underwent CD were generally older and exhibited a higher prevalence of obesity, hypertensive disorders (including chronic hypertension, gestational hypertension, or preeclampsia), and diabetes mellitus (both pre-gestational and gestational). Furthermore, women in the CD groups were more likely to have undergone fertility treatments. Nulliparity was twice as common in the NPL groups compared to the VD group.

Table 2 presents selected pregnancy and perinatal outcomes across the study groups. The highest mean birthweight was observed in the NPL2 group, followed by the NPL1 and VD groups. Correspondingly, the incidence of low birth weight (LBW) infants was highest in the VD group and lowest in the NPL2 group. Low Apgar scores were more frequent among neonates born via CD compared to those delivered vaginally, especially in the NPL2 group; however, mean umbilical arterial pH at birth was comparable across all groups. The incidence of postpartum hemorrhage (PPH) was highest in the NPL2 group.

Table 3 outlines selected infectious morbidities among offspring in the different groups. Compared to VD, children born via CD due to NPL1 exhibited the highest rates of asthma and obstructive sleep apnea (OSA). The overall rate of respiratory-related hospitalizations was highest among offspring in the NPL1 group. Cesarean delivery due to NPL1 was associated with 2.0 additional cases of respiratory morbidity per 100 births (absolute risk 2.0% compared to VD), and cesarean delivery due to NPL2 was associated with 0.2 additional cases of respiratory morbidity per 100 births (absolute risk 0.2% compared to VD). 

Median follow up time were 2934 days for the VD group; 4196 days for the NPL1 group and 2199 days for the NPL2 group. The ICD-9 codes used for offspring respiratory morbidity are detailed in the Supplementary Table (Table S1).

**Figure 1:**
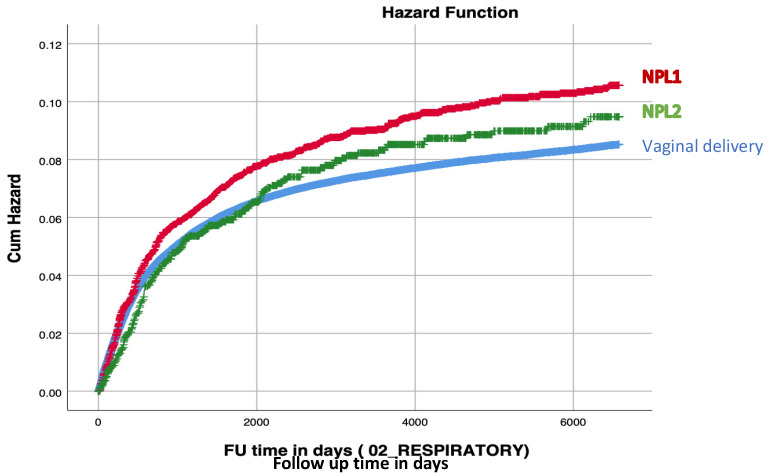
Kaplan-Meier survival curve demonstrating the cumulative incidence of respiratory-related hospitalizations among study groups (Log-rank < 0.001).

The Kaplan–Meier survival curve (Figure 1) demonstrated a higher cumulative incidence of respiratory-related hospitalizations, with a pattern consistent with increasing incidence corresponding to decreasing fetal exposure to vaginal flora. This trend was observed across VD, NPL2 and NPL1 with the latter exhibiting the highest incidence (log-rank, *p* < 0.001).

The multivariable Cox regression model presented in Table 4, constructed to adjust for maternal age, nulliparity, maternal obesity, smoking, use of fertility treatments, diabetes mellitus, hypertensive disorders, gender, ethnicity, birthweight and child’s year of birth, identified that CD due to NPL1 is an independent risk factors for increased long-term respiratory morbidity in offspring (adjusted hazard ratio (aHR) 1.15). In contrast, NPL2 and VD groups did not demonstrate a significant difference in this risk.

## 4. Discussion

Our study adds to the expanding body of literature highlighting the importance of exposure to maternal vaginal microbiota during labor in influencing long-term respiratory outcomes in offspring. We observed a significant association between delivery mode and the risk of respiratory-related hospitalizations in children through 18 years of age. Specifically, those born vaginally with the greatest exposure to maternal vaginal flora, had the lowest risk of long-term respiratory morbidity compared to those with presumed partial exposure (NPL2) or minimal exposure (NPL1). Moreover, the observed graded association between delivery mode and the stage of labor to respiratory outcomes highlights the importance of maternal flora exposure, even in the context of cesarean delivery.

Passage through the birth canal exposes the infant to maternal vaginal and intestinal microbiota, which play a crucial role in immune system development and may influence respiratory health. Those delivered vaginally are exposed to vaginal and fecal microbiota, facilitating colonization by beneficial microbes like *Lactobacillus*, *Bacteroides* and *Bifidobacterium*. In contrast, cesarean-delivered infants lack direct maternal microbial contact and tend to acquire microbes from maternal skin, hospital environments, or staff [2].

Specifically, the airway microbiota of offspring after CD is characterized by distinct differences compared to those delivered vaginally. The initial airway microbiota in CD infants is less diverse and more likely to be dominated by skin-associated bacteria such as *Staphylococcus*. Also, infants born by CD exhibit a delayed development of their respiratory microbiota. Studies show that there is a reduced colonization with health-associated commensals such as *Corynebacterium* and *Dolosigranulum* in CD infants, which may influence respiratory health later in life [5,18,19].

Additionally, the differences in gut and airway microbiota between vaginally delivered and cesarean-born infants have been linked to variations in immune responses and potential long-term respiratory outcomes. Many studies indicate that CD infants show increased prevalence of skin and gut bacteria such as *Staphylococcus* and *Clostridium*. Emerging evidence suggests that these microbial alterations linked to CD are associated with chronic health issues, including obesity, metabolic disorders, inflammatory bowel disease, atopy, and asthma [2]. Specifically, children born after elective CD were shown to suffer from neonatal respiratory complications, partly due to the lack of exposure to maternal microbiota during vaginal delivery as they are deprived of the physiological processes of labor [20]. Several studies have shown that infants born via elective CD experience increased rates of respiratory complications compared to those delivered vaginally. Hansen et al. reported that elective CD performed at term significantly elevated the risk of respiratory conditions, including transient tachypnea of the newborn (TTN), respiratory distress syndrome (RDS), and persistent pulmonary hypertension of the newborn (PPHN) [21]. Baumfeld et al. also reported that elective CD at term is associated with a higher long-term risk of respiratory morbidity, including conditions like asthma and obstructive sleep apnea, compared to vaginal delivery [9]. This association persisted even after adjusting for confounders such as maternal age, gestational age, and birthweight.

The findings of this study reveal significant differences in respiratory morbidity among children born via CD due to NPL1 compared to the VD and those delivered for NPL2. Specifically, infants born via CD due to NPL1 exhibited the highest rates of asthma and OSA, as well as the highest overall rates of respiratory-related hospitalizations. NPL1 shares similarities with elective cesarean delivery in that both scenarios result in suboptimal exposure to maternal vaginal flora, disrupting the normal development of the infant’s microbiome, which is essential for immune system maturation and respiratory health.

Attempts of vaginal seeding to expose the infant to maternal vaginal microbiota and restore the neonatal microbiome to resemble that of vaginally delivered infants, typically after cesarean delivery, have been investigated in recent years. While current evidence demonstrates that vaginal seeding can partially restore the microbiome composition of cesarean-delivered infants [22], improvements in health outcomes, including neonatal respiratory outcomes, are limited and inconsistent; no studies have demonstrated a direct benefit of vaginal seeding on neonatal respiratory outcomes [23,24]. As such, current guidelines emphasize that vaginal seeding should not be performed outside of research protocols due to concerns about infectious risks (e.g., group B *Streptococcus*, HSV, HIV) and lack of proven clinical benefit [25].

While microbiome-related mechanisms may explain some of our results, additional alternative pathways also deserve consideration. During labor, there is a surge in catecholamines (notably norepinephrine and epinephrine) and activation of the fetal hypothalamic–pituitary–adrenal (HPA) axis, leading to increased endogenous glucocorticoid (cortisol) production, which facilitate key processes in pulmonary adaptation in the transition from intrauterine to extrauterine life [26]. Lung fluid clearance during labor is primarily mediated by a switch in the distal lung epithelium from secretion to absorption, driven by the surge in catecholamines and endogenous glucocorticoids [27]. These hormones upregulate epithelial sodium channels (ENaC) and Na-K-ATPase in alveolar cells, promoting active sodium reabsorption from the alveolar space into the interstitium. They also stimulate alveolar cells to synthesize and secrete surfactant lipids and proteins which is essential for reducing alveolar surface tension. The magnitude of these effect correlates with the degree of birth stress and catecholamine surge, as evidenced in neonates exposed to labor compared to those delivered by elective cesarean section [28].

Mechanical factors, such as fetal thoracic and abdominal muscle contractions during labor, also contribute to the expulsion of lung fluid through the trachea [29], but hormonal regulation is the dominant mechanism for alveolar fluid clearance. These physiological processes of lung fluid clearance and surfactant production that occur during labor may contribute to the observed differences independent of microbial exposure.

The main strength of this study is its large cohort and long-term follow-up period. As previously noted, SUMC serves as the only medical center in the region; therefore, most women who deliver at our institution are also likely to return there for their children’s medical care when needed. This regional continuity enabled us to leverage linked perinatal and pediatric databases to track offspring from birth through 18 years of age and to capture a broad spectrum of respiratory conditions occurring throughout infancy, childhood, and adolescence. Second, the study method used to focus specifically on intra-partum CD for reasons of labor dystocia, unrelated to other possible intra-partum complications, is probably the first large cohort study that stratified dystocia by labor stage, which allows for an innovative analysis of conditions that were not investigated before in this context.

The major limitation of this study is the retrospective nature of the study design as it introduces inherent biases that limit the ability to establish causality. The original partograms of laboring women were unavailable for analysis; therefore, detailed data on cervical dilation and labor duration could not be assessed. Nonetheless, the definitions of the first and second stages of labor were uniform and based on universally accepted criteria.

We also acknowledge that the biological plausibility of our findings is only implied by the theory of exposure to maternal vaginal flora and the true exposure levels or vaginal microbiome was not directly measured in this study. Future research investigating vaginal microbiota and comparing offspring long-term outcomes is encouraged.

Another limitation of this study is the substantial imbalance between the number of vaginal deliveries and cesarean deliveries. This disparity reflects real-world obstetric practice but may nonetheless introduce bias in the interpretation of the results, particularly for less frequent outcomes.

Also, it is acknowledged that women who progress to second-stage arrest fundamentally differ from those arrested in the first stage, representing different labor physiology and potentially different underlying maternal–fetal characteristics. However, we have applied a meticulous multivariable model to account for many variables that differed among groups and found that the heightened risk for NPL1 infants remained statistically significant.

Additionally, the respiratory morbidity of children in this study was based on hospitalization records and these are probably only the severe cases of respiratory illness that necessitated hospitalization. It is assumed that most respiratory diseases in children are primarily treated in an ambulatory setting and would not be covered in this study. Nevertheless, having found an association between vaginal flora exposure and respiratory morbidity, as reflected in severe cases of hospitalizations, suggests that the true magnitude may be even greater in milder cases of respiratory morbidity. Additional studies incorporating data of respiratory morbidity in non-hospitalized patients would validate our findings and elucidate the underlying mechanisms.

Finally, as a single-center study conducted in southern Israel with specific population demographics, the results may not apply to other settings with different ethnic compositions, healthcare systems, or obstetric practices.

## 5. Conclusions

As CD rates continue to rise globally, understanding its potential long-term impact on offspring health is essential for both clinicians and patients. Healthcare providers frequently navigate decisions regarding the most appropriate mode of delivery, and the findings of this study contribute valuable evidence to inform clinical decision-making. By highlighting the potential long-term respiratory benefits associated with vaginal birth, this research supports more informed discussions between healthcare professionals and expectant mothers. While elective CD may be necessary for specific maternal or fetal indications, these findings emphasize the importance of considering its broader implications on child health. In cases where trial of labor after cesarean (TOLAC) is an option, counseling should include not only maternal risks and benefits but also the potential long-term respiratory outcomes for the child. Ultimately, integrating this knowledge into obstetric decision-making may help optimize both immediate and long-term health outcomes for mothers and their offspring.

## Figures and Tables

**Table 1:** Demographic and baseline maternal characteristics among all study groups.

Characteristic	Vaginal Delivery (*n* = 306,434)	NPL1 (*n* = 5149)	NPL2 (*n* = 2199)	*p* Value
Maternal age (years, ± SD)	27.9 ± 5.7	29.2 ± 6.0	28.2 ± 5.6	<0.001
Nulliparity (%)	73,310 (23.9)	2177 (42.3)	1261 (57.3)	<0.001
Fertility treatment ^a^ (%)	3939 (1.3)	228 (4.4)	72 (3.3)	<0.001
Obesity (%)	2595 (0.8)	176 (3.4)	49 (2.2)	<0.001
Smoking (%)	2040 (0.7)	49 (1.0)	10 (0.5)	0.021
Hypertensive disorders ^b^ (%)	11,797 (3.8)	640 (12.4)	191 (8.7)	<0.001
Diabetes mellitus ^c^ (%)	12,257 (4.0)	530 (10.3)	187 (8.5)	<0.001

^a^ Including all artificial reproductive techniques: ovulation induction and in vitro fertilization (IVF). ^b^ Including chronic hypertension, gestational hypertension and pre-eclampsia. ^c^ Including pre-gestational and gestational diabetes mellitus.

**Table 2:** Obstetrical complications and perinatal outcomes by mode of delivery and indication for CD.

Outcome	Vaginal Delivery (*n* = 306,434)	NPL1 (*n* = 5149)	NPL2 (*n* = 2199)	*p* Value
Gestational age (weeks ± SD)	39.1 ± 1.8	39.6 ± 1.5	39.6 ± 1.4	<0.001
Preterm delivery < 37 (%)	16,990 (5.5)	179 (3.5)	43 (2.0)	<0.001
Birthweight (grams, mean ± SD)	3214 ± 489	3364 ± 495	3455 ± 448	<0.001
Low birthweight < 2500 g (%)	17,524 (5.7)	235 (4.6)	34 (1.5)	<0.001
PH (±SD)	7.35 ± 0.07	7.30 ± 0.08	7.27 ± 0.09	0.237
5 min Apgar < 7 (%)	1066 (0.4)	69 (1.3)	55 (2.5)	<0.001
Post-partum hemorrhage (%)	1857 (0.6)	15 (0.3)	18 (0.8)	0.006

**Table 3:** Offspring respiratory-related morbidity according to delivery mode and CD indication.

Respiratory Morbidity	Vaginal Delivery (*n* = 306,434)	NPL1 (*n* = 5149)	NPL2 (*n* = 2199)	*p* Value
Asthma (%)	5819 (1.9)	142 (2.8)	47 (2.1)	<0.001
Structural—Emphysema (%)	55 (0.0)	3 (0.1)	1 (0.0)	0.074
Bronchiectasis (%)	3902 (1.3)	96 (1.9)	45 (2.0)	<0.001
Pneumonitis (%)	264 (0.1)	7 (0.1)	1 (0.0)	0.390
Pleural disease (%)	459 (0.1)	14 (0.3)	1 (0.0)	0.036
Obstructive sleep apnea (OSA) (%)	3931 (1.3)	107 (2.1)	28 (1.3)	<0.001
Total respiratory-related Hospitalizations (%)	21,215 (6.9)	459 (8.9)	156 (7.1)	<0.001

**Table 4:** A multivariable Cox model assessing the association between delivery mode and CD indication and the risk for offspring hospitalization with respiratory-related morbidity.

	Adjusted Hazards Ratio (aHR)	95% Confidence Interval	*p* Value
CD for NPL1	1.15	1.09–1.21	<0.001
CD for NPL2	1.06	0.98–1.15	0.140
Vaginal delivery	1 (reference)	-	-
Maternal age	0.99	0.99–0.99	<0.001
Birthweight	1.00	1.00–1.00	<0.001
Ethnicity	0.80	0.79–0.81	<0.001
Child birth year	1.04	1.04–1.04	<0.001
Gender	1.36	1.34–1.37	<0.001
Use of fertility treatments	1.02	0.96–1.07	0.459
Nulliparity	0.93	0.91–0.94	<0.001
Obesity	0.96	0.89–1.04	0.388
Smoking	1.74	1.62–1.87	<0.001
Hypertensive disorders	1.02	0.98–1.05	0.255
Diabetes mellitus	1.18	1.14–1.22	<0.001

## Data Availability

The raw data supporting the conclusions of this article will be made available by the authors on request.

## Supplementary Materials

The following supporting information can be downloaded at: https://www.mdpi.com/article/10.3390/jcm15051827/s1, Table S1: ICD-9 codes for respiratory morbidity investigated in this study.

## Abbreviations

The following abbreviations are used in this manuscript:

CD	Cesarean delivery
NPL	Non-progressive labor
VD	Vaginal delivery
